# Poring over furrows

**DOI:** 10.7554/eLife.27933

**Published:** 2017-05-31

**Authors:** Skylar ID Fisher, H Criss Hartzell

**Affiliations:** 1 Department of Cell Biology, Emory University School of Medicine, Atlanta, United States; 1 Department of Cell Biology, Emory University School of Medicine, Atlanta, United Statescriss.hartzell@emory.edu

**Keywords:** Ligand Gated Ion Channels, cryo-electron microscopy, patch-clamp electrophysiology, ion permeation, Mouse

## Abstract

Cryo-electron microscopy reveals the structure of a chloride channel that is closely related to a protein that transports lipids.

**Related research article** Paulino C, Neldner Y, Lam AKM, Kalienkova V, Brunner JD, Schenck S, Dutzler, R. 2017. Structural basis for anion conduction in the calcium-activated chloride channel TMEM16A. *eLife* **6**:e26232. doi: 10.7554/eLife.26232

Proteins known as calcium-activated chloride channels have a central role in processes as diverse as regulating blood pressure in mammals and closing the Venus flytrap (Hartzell et al., 2005). In 2008, it was discovered that the archetypal forms of these channels are encoded by a gene called *TMEM16A* (also known as *ANO1*; Caputo et al., 2008; Schroeder et al., 2008; Yang et al., 2008). Since this discovery, hundreds of papers have been published about these channels, providing important insights into the relationship between their structure and function. However, relatively little was known about the structure of the channels at the atomic level. Now, in eLife, Raimund Dutzler at the University of Zurich and colleagues – including Cristina Paulino as first author – report that they have used cryo-electron microscopy (cryo-EM) to obtain a near-atomic resolution structure of a mouse TMEM16A channel (Paulino et al., 2017).

The TMEM16 proteins are also known as anoctamins because it was first thought that all the proteins in the TMEM16A family contained eight (octa) transmembrane domains and had pores that allowed chloride ions and other anions to traverse membranes. However, the use of this name has been controversial because many of the TMEM16 proteins are not, in fact, anion channels, despite the similarity in their amino acid sequences. Rather, most of them are phospholipid scramblases that passively transport phospholipid molecules between the two lipid layers of cell membranes (Suzuki et al., 2010; Bevers and Williamson, 2016).

Phospholipids are amphipathic: they contain a hydrophilic ('water-loving') head attached to a hydrophobic ('water-hating') tail. When they assemble into membranes, the tails of the phospholipids in the outer layer orient towards the tails of the phospholipids in the inner layer so that the membrane has a hydrophobic core and hydrophilic surfaces. The Dutzler laboratory revealed how TMEM16 scramblases transport lipids between layers when they solved the X-ray structure of a fungal phospholipid scramblase known as nhTMEM16 (Brunner et al., 2014). This structure showed that these scramblases are made of pairs (dimers) of TMEM16 molecules. Each 'subunit' in the dimer has an unusual hydrophilic furrow on its surface that allows the heads of the phospholipids to move from one layer to the other while their tails remain in the hydrophobic core of the membrane.

The X-ray structure of nhTMEM16 sparked many questions about the differences between the structures of the scramblases and the ion channels. Unlike the scramblases, which transport amphipathic phospholipids, TMEM16A channels support the passage of small hydrophilic anions. It was initially hypothesized that TMEM16A subunits might form dimers with their hydrophilic grooves facing one another to create a single hydrophilic pore in the center (Brunner et al., 2014). However, this hypothesis was called into question by studies that demonstrated that TMEM16A dimers have two separate pores (Jeng et al., 2016; Lim et al., 2016). We suggested that the pore is composed of both proteins and lipids (Whitlock and Hartzell, 2016), but this model is now challenged by the new structure of mouse TMEM16A.

Paulino et al. now show that the mouse TMEM16A channel, like the nhTMEM16 scramblase, forms dimers with each subunit having 10 helices that span the membrane. Instead of being an open furrow, the ion-conducting pore in the channel is enclosed by protein along most of its length. This is due to a structural rearrangement that brings the two helices that form the edges of the furrow in nhTMEM16 together in TMEM16A to enclose the pore (Figure 1). The pore is shielded from the hydrophobic core of the membrane for about two-thirds the distance across the membrane while the remainder is open to the lipids and cytoplasm.

**Figure 1.**
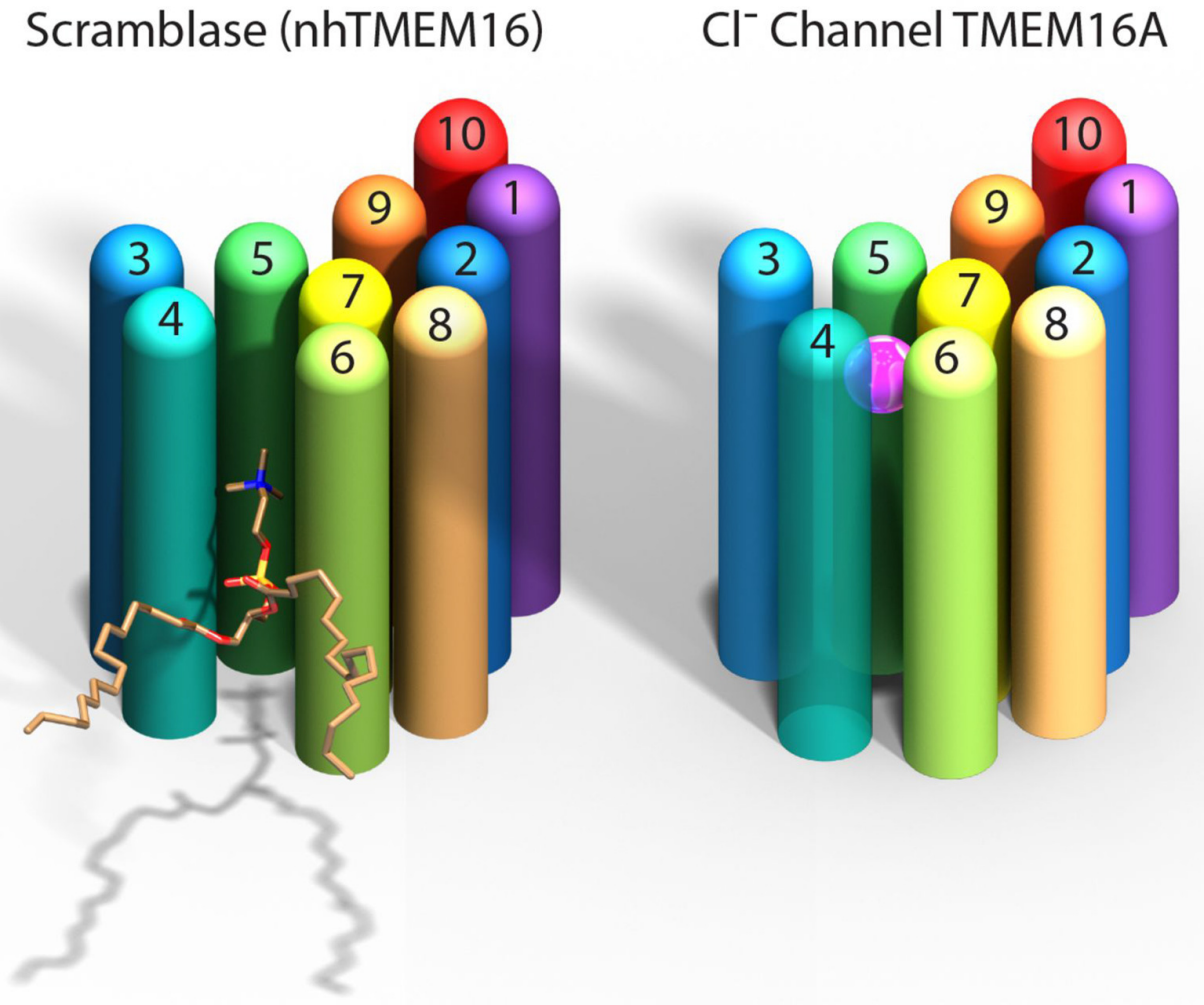
Comparison of a TMEM16 scramblase and a TMEM16 chloride channel. The fungal nhTMEM16 protein (left), which acts as a lipid scramblase, and the mouse TMEM16A protein (right), which operates as a chloride ion channel, both contain 10 alpha helices (shown here as colored cylinders) embedded in the membrane. Only one subunit of the dimer is shown. The lower end of each protein faces into the cell, while the upper end faces outwards. While there are small differences in the relative locations of most of the helices, the biggest difference between the two proteins is the position of helix 4: in nhTMEM16 helix 4 is quite close to helix 3, which leads to the formation of a furrow by helices 4, 5 and 6; in TMEM16A helix 4 is further away from helix 3, and closer to helix 6, which leads to the formation of a narrow pore by helices 3–7. The furrow is able to transport lipids such as phosphatidylcholine (shown here), whereas the pore can transport chloride ions (pink sphere).

This model confirms the double-barreled nature of TMEM16A (Jeng et al., 2016; Lim et al., 2016): each subunit of the dimer has its own pore with the interface between the two formed largely by just one helix in each subunit. Confidence in the overall structural model is provided by experiments using mutagenesis to substitute positively charged amino acids in the pore with neutral ones. The effects of these substitutions are consistent with the idea that altering the charge of the pore will affect the ability of anions to move through the pore.

Several key questions remain. It is very likely that the TMEM16A structure described here is the conformation of the protein when it is bound to calcium ions because it was purified in the presence of high levels of calcium ions. However, it was not possible to determine the exact locations of individual amino acids within the pore, so the details of the geometry of the pore remain uncertain. Since TMEM16A readily inactivates in the presence of high levels of calcium ions (Lim et al., 2016), this structure could conceivably represent a state that does not conduct ions.

The narrowest part of the pore in the cryo-EM structure is estimated to be 3.6 angstroms wide, which is exactly the diameter of a chloride ion. This raises the question of how the pore could accommodate the passage of larger ions, such as iodide and tricyanomethanide, which are also known to be able to move through TMEM16A channels (Qu and Hartzell, 2000; Schroeder et al., 2008). In spite of this ambiguity, the findings of Paulino et al. are an essential step forward in understanding how chloride ions pass through TMEM16 channels and the evolutionary relationships between the members of this protein family.

## References

[bib1] Bevers EM, Williamson PL (2016). Getting to the outer leaflet: physiology of phosphatidylserine exposure at the plasma membrane. Physiological Reviews.

[bib2] Brunner JD, Lim NK, Schenck S, Duerst A, Dutzler R (2014). X-ray structure of a calcium-activated TMEM16 lipid scramblase. Nature.

[bib3] Caputo A, Caci E, Ferrera L, Pedemonte N, Barsanti C, Sondo E, Pfeffer U, Ravazzolo R, Zegarra-Moran O, Galietta LJ (2008). TMEM16A, a membrane protein associated with calcium-dependent chloride channel activity. Science.

[bib4] Hartzell C, Putzier I, Arreola J (2005). Calcium-activated chloride channels. Annual Review of Physiology.

[bib5] Jeng G, Aggarwal M, Yu WP, Chen TY (2016). Independent activation of distinct pores in dimeric TMEM16A channels. The Journal of General Physiology.

[bib6] Lim NK, Lam AK, Dutzler R (2016). Independent activation of ion conduction pores in the double-barreled calcium-activated chloride channel TMEM16A. The Journal of General Physiology.

[bib7] Paulino C, Neldner Y, Lam AKM, Kalienkova V, Brunner JD, Schenck S, Dutzler R (2017). Structural basis for anion conduction in the calcium-activated chloride channel TMEM16A. eLife.

[bib8] Qu Z, Hartzell HC (2000). Anion permeation in Ca^2+^-activated Cl^-^ channels. The Journal of General Physiology.

[bib9] Schroeder BC, Cheng T, Jan YN, Jan LY (2008). Expression cloning of TMEM16A as a calcium-activated chloride channel subunit. Cell.

[bib10] Suzuki J, Umeda M, Sims PJ, Nagata S (2010). Calcium-dependent phospholipid scrambling by TMEM16F. Nature.

[bib11] Whitlock JM, Hartzell HC (2016). A pore Idea: the ion conduction pathway of TMEM16/ANO proteins is composed partly of lipid. Pflügers Archiv - European Journal of Physiology.

[bib12] Yang YD, Cho H, Koo JY, Tak MH, Cho Y, Shim WS, Park SP, Lee J, Lee B, Kim BM, Raouf R, Shin YK, Oh U (2008). TMEM16A confers receptor-activated calcium-dependent chloride conductance. Nature.

